# A practical nomogram for predicting amputation rates in acute compartment syndrome patients based on clinical factors and biochemical blood markers

**DOI:** 10.1186/s12891-023-06746-7

**Published:** 2023-08-09

**Authors:** Donglei Wei, Jianwen Cheng, Yage Jiang, Nanchang Huang, Jianhui Xiang, Junfeng Li, Hui Wang, Wei Su, Jinmin Zhao

**Affiliations:** 1 Department of Traumatology and Hand Surgery, The First Affiliated Hospital of Guangxi Medical University, No. 6 Shuangyong Road, Nanning, Guangxi China; 2 Guangxi Key Laboratory of Regenerative Medicine, Orthopaedic Department, The First Affiliated Hospital of Guangxi Medical University, No. 6 Shuangyong Road, Nanning, Guangxi China; 3 Department of Anesthesiology, The First Affiliated Hospital of Guangxi Medical University, No. 6 Shuangyong Road, Nanning, Guangxi China

**Keywords:** Acute compartment syndrome, Amputation, Predictors, Nomogram

## Abstract

**Background:**

Amputation is a serious complication of acute compartment syndrome (ACS), and predicting the risk factors associated with amputation remains a challenge for surgeons. The aim of this study was to analyze the risk factors for amputation in patients with ACS and develop a nomogram to predict amputation risk more accurately.

**Methods:**

The study population consisted of 143 patients (32 in the amputation group and 111 in the limb preservation group) diagnosed with ACS. LASSO and multivariate logistic regression were used to screen predictors and create a nomogram. The model’s accuracy was assessed by receiver operating characteristic (ROC) curves, C-index, calibration curves, and decision curve analysis (DCA).

**Results:**

The predictors included cause of injury, vascular damage, shock, and fibrinogen in the nomogram. The C-index of the model was 0.872 (95% confidence interval: 0.854–0.962), and the C-index calculated by internal validation was 0.838. The nomogram’s area under the curve (AUC) was 0.849, and the calibration curve demonstrated a high degree of agreement between the nomogram’s predictions and actual observations. Additionally, the DCA indicated good clinical utility for the nomogram.

**Conclusion:**

The risk of amputation in ACS patients is associated with the cause of injury, vascular damage, shock, and fibrinogen. Our nomogram integrating clinical factors and biochemical blood markers enables doctors to more conveniently predict the risk of amputation in patients with ACS.

## Introduction

Acute Compartment Syndrome (ACS) is a severe and frequent complication in traumatic orthopedics that commonly occurs in cases of bilateral bone fractures, such as those of the forearm and lower leg. ACS is caused by a decrease in local circulatory perfusion that leads to tissue ischemia and metabolic product accumulation within the fascial compartment, which results in increased interfacial pressure and irreversible muscle and nerve damage [1, 2]. Early diagnosis and prompt emergency fasciotomy decompression are effective treatments for ACS. However, failed or delayed fasciotomy decompression may lead to complications such as ischemic muscle contracture, permanent motor and sensory deficits, infection, bone discontinuity, and in severe cases, amputation or death [3]. Patients who have failed, delayed, or missed fasciotomy decompression of ACS have been reported to have a fourfold higher mortality rate and a twofold higher amputation rate than those who received early complete fasciotomy decompression [4].

The risk factors linked to the development of amputation in patients with ACS have not been precisely defined. Zhang et al. discovered that diabetes mellitus, lack of intercompartmental pressure measurements, long partial thromboplastin time, and low albumin levels were risk factors associated with amputation in 546 ACS patients [5]. Other related studies have found that elevated creatine kinase levels, smoking, open fractures, trauma severity, and high-energy injuries are associated with amputation in patients with ACS [6, 7]. The incidence of serious complications of ACS is high, with a mortality rate of 2–15% and an amputation rate of 10–21% according to published studies [5, 8, 9]. Hence, it is crucial to identify high-risk patients early and implement specific interventions to reduce the risk of amputation in patients with ACS.

Nomograms, which visually combine multiple risk factors to predict the likelihood of an event, are widely used to predict medical diseases [10–12]. However, no studies have been conducted using nomograms to predict the risk of amputation in patients with ACS. Therefore, the aim of our study was to investigate the incidence of amputation and its associated risk factors and to develop a nomogram for predicting the risk of amputation in patients with ACS.

## Methods

### Patients

A total of 143 patients with ACS were retrospectively followed up from January 2010 to May 2020. All participants provided informed consent for this study, which was approved by the Medical Ethics Committee (ethical approval number: NO.2022-KY-(055)) and complied with the World Medical Association Declaration of Helsinki. To be included in the study, patients had to meet the following criteria: (1) a diagnosis of ACS or Volkmann contracture and (2) osteofascial compartment dissection and decompression. The exclusion criteria were as follows: (1) chronic fascial compartment syndrome; (2) crush syndrome; (3) incomplete patient history; and (4) presentation in combination with a blood-related disease (such as hemophilia, leukemia, thalassemia, etc.). Osteofascial compartment dissection and decompression is a surgical procedure performed promptly upon the diagnosis of ACS to relieve the pressure in the affected compartment. The surgeon begins by making an incision along the length of the affected compartment, followed by careful dissection of the fascia surrounding the muscles to release any constricting bands or fascial defects. During the procedure, the surgeon continuously monitors the compartment pressure to ensure that it remains within safe levels.

### Data collection

In this study, we collected patient data through an electronic medical record system, which included demographic information (age, sex, occupation, ethnicity, residence, marital status, smoking, drinking, surgical and trauma history, previous history of blood transfusion, temperature, heart rate, etc.), clinical characteristics (amputation, cause of injury, fracture, vascular damage, shock, and multiorgan dysfunction (MODS)), treatment status (blood transfusion, surgeon’s level, first hospital level, injury-to-care interval), and biochemical blood markers (white blood cells (WBC), platelets (PLT), hemoglobin (HGB), neutrophil percentage (NEU), total bilirubin, total protein, albumin, aspartate aminotransferase (AST), alanine aminotransferase (ALT), prealbumin (PA), urea, potassium, sodium, chloride, prothrombin time (PT), and fibrinogen (FIB)). The blood biochemical markers were obtained at the time of the first ACS diagnosis and were classified according to reference values. Patients were divided into two groups based on whether they underwent amputation, i.e., the limb preservation group and the amputation group. In addition, the Mangled Extremity Severity Score (MESS) is a grading system that evaluates bone and soft tissue injury, shock, ischemia, and age to assess the severity of extremity trauma and predict the need for amputation of the affected limb(s) [13, 14]. We assessed the severity of extremity trauma in two patient groups by evaluating MESS.

### Statistical analysis

Data were analyzed using R software version 4.1.1 for Windows. The least absolute shrinkage and selection operator (LASSO) method was used to screen for risk factors with the best predictive characteristics [15]. A plot of the coefficient profile against the log (lambda) sequence was made and the minimum criteria and their 1-SE values (the 1-SE criteria) were used to construct dotted vertical lines at optimal values. Variables that were statistically significant (p < 0.05) from the LASSO regression model were included in the multivariate logistic regression analysis. Subsequently, variables with statistical significance from the multivariate logistic regression analysis were selected for the subsequent analysis, and a predictive nomogram was developed. The predictive power and performance of the nomogram were assessed using the C-index, the area under the ROC curve, and the calibration curve. In the calibration curve, the x-axis shows the predicted risk of amputation and the y-axis shows the actual risk of amputation. The diagonal line with dots shows what a perfect model would predict and the solid line shows how well the nomogram works. A better prediction is made when the solid line is closer to the diagonal dotted line. The C-index and AUC values were interpreted as low accuracy (< 0.5), moderate accuracy (0.5–0.7), high accuracy (0.7–0.9), and extreme accuracy (> 0.9) [16]. To evaluate the clinical utility of the nomogram, we used DCA curves to estimate the net benefit and threshold probability. The p-value was set to p < 0.05, indicating statistical significance. The nomogram’s internal validation was performed using bootstrap validation to calculate the corrected C-index based on 1000 bootstrap resamples, and it was used to validate the amputation risk nomogram.

## Results

### Patient characteristics

**Fig. 1:**
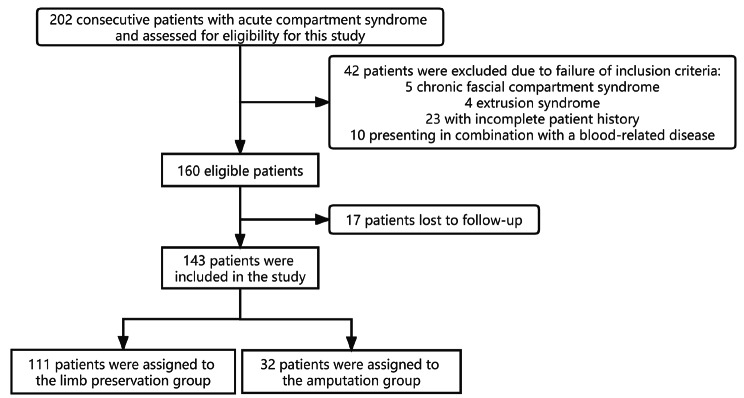
Flow diagram of the patient selection

After applying inclusion and exclusion criteria, a total of 143 patients with ACS were included in this study (Fig. 1). The patient cohort comprised 116 males and 27 females, with a mean age of 35.01 ± 18.20 years. The cohort was further divided into two groups based on the treatment received: a limb-preserving group (111 cases) and an amputation group (32 cases). The Mangled Extremity Severity Score (MESS) was 4.21 ± 1.03 in the limb-preserving group and 5.04 ± 2.14 in the amputation group (p = 0.0028). The study’s demographic data mainly consisted of Zhuang and Han patients, who accounted for 47% and 48% of the study population, respectively, while 5% of the patients were from other ethnic backgrounds. The majority of the patients (83%) came from rural areas. The primary causes of injury were traffic accidents (56%), falls (25%), electric shock (4%), and vascular disease (5%). Among the cases included in the study, 50 involved upper extremities and 93 involved lower extremities. Vascular damage, including popliteal, anterior tibial, posterior tibial, brachial, radial, and ulnar arteries, occurred in 41 cases (29%), and 56% of the amputation cases included vascular damage. Hemorrhagic shock symptoms were present in 22 patients. All blood biochemical markers were classified according to the reference values. Univariate analysis demonstrated that the cause of injury, vascular damage, white blood cell count (WBC), hemoglobin (HGB), total bilirubin, total protein, albumin, aspartate transaminase (AST), alanine transaminase (ALT), prealbumin, uric acid, and fibrinogen (FIB) levels were significantly associated with the risk of amputation (p < 0.05). Table 1 presents all clinical and biochemical data for both study groups.

**Table 1:** Univariate analysis of demographic characteristics and blood biochemical markers

Characteristics	n(%)			P
	Limb preservation (n = 111)	Amputation (n = 32)	Total (n = 143)	
Gender				0.119
Male	87(78)	29(91)	116(81)	
Female	24(22)	3(9)	27(19)	
Age(years)				0.469
> 18	31(28)	3(9)	34(24)	
18–60	66(59)	29(91)	95(66)	
> 60	14(13)	0(0)	14(10)	
Race				0.264
Han	52(47)	17(53)	69(48)	
Zhuang	55(50)	12(38)	67(47)	
Others	4(4)	3(9)	7(5)	
Residence				0.83
Rural	92(83)	26(81)	118(83)	
City	19(17)	6(19)	25(17)	
Profession				0.129
Light manual labor	56(50)	21(66)	77(54)	
Heavy physical labor	55(50)	11(34)	66(46)	
Marriage				0.282
yes	57(51)	21(66)	78(55)	
no	52(47)	10(31)	62(43)	
Divorced	2(2)	1(3)	3(2)	
Smoking				0.382
yes	17(15)	7(22)	24(17)	
no	94(85)	25(78)	119(83)	
Drinking				0.712
yes	13(12)	3(9)	16(11)	
no	98(88)	29(91)	127(89)	
Surgical history				0.285
yes	19(17)	3(9)	22(15)	
no	92(83)	29(91)	121(85)	
Trauma history				0.929
yes	11(10)	3(9)	14(10)	
no	100(90)	29(91)	129(90)	
Pre-blood transfusion				0.49
yes	7(6)	1(3)	8(6)	
no	104(94)	31(97)	135(94)	
Cause injury				0.003
Traffic	55(50)	25(78)	80(56)	
Fall	34(31)	2(6)	36(25)	
Electric or thermal	3(3)	3(10)	6(4)	
Vascular disease	19(17)	2(6)	21(15)	
First hospital				0.086
District hospitals	58(52)	13(41)	71(50)	
County-level hospitals	9(8)	7(22)	16(11)	
City or provincial-level hospitals	44(40)	12(38)	56(39)	
Intervals				0.26
6 h	29(26)	5(16)	34(24)	
6 h-1d	55(50)	21(66)	76(53)	
> 1d	27(24)	6(19)	33(23)	
Transfer				0.089
yes	81(73)	28(88)	109(76)	
no	30(27)	4(12)	34(24)	
T(°C)				
> 37.5	105(95)	25(78)	130(91)	
>=37.5	6(5)	7(22)	13(9)	
HR				0.164
> 100	78(70)	18(56)	96(67)	
100–120	21(19)	12(38)	33(23)	
>=120	12(11)	2(6)	14(10)	
Body parts				0.078
Upper	43(39)	7(22)	50(35)	
Lower	68(61)	25(78)	93(65)	
Fracture				0.721
yes	69(62)	21(66)	90(63)	
no	42(38)	11(34)	53(37)	
Vascular damage				< 0.001
yes	23(21)	18(56)	41(29)	
no	88(79)	14(44)	102(71)	
Surgeon				0.634
Resident	37(33)	13(41)	50(35)	
Attending	34(31)	11(34)	45(31)	
Deputy Chief	9(8)	1(3)	10(7)	
Chief	31(28)	7(22)	38(27)	
Shock				0.087
yes	14(13)	8(25)	22(15)	
no	97(87)	24(75)	121(85)	
Blood transfusion				0.139
yes	24(22)	11(34)	35(24)	
no	87(78)	21(66)	108(76)	
MODS				0.511
yes	4(4)	2(6)	6(4)	
no	107(96)	30(94)	137(96)	
WBC(10^9^/L)				0.002
< 3.5 or > 9.5	61(55)	25(78)	86(60)	
3.5–9.5	50(45)	7(22)	57(40)	
HGB (g/L)				< 0.001
< 120	53(48)	26(81)	79(55)	
>=120	58(52)	6(19)	64(45)	
PLT(10^9^/L)				0.202
< 125 or > 350	33(30)	13(41)	46(32)	
125–350	78(70)	19(59)	97(68)	
NEU(%)				0.441
< 0.4 or > 0.75	57(51)	26(81)	83(58)	
0.4–0.75	54(49)	6(19)	60(42)	
Total bilirubin (umol/L)				< 0.001
< 3.4 or > 20.5	20(18)	6(19)	26(18)	
3.4–20.5	91(82)	26(81)	117(82)	
Total protein (g/L)				< 0.001
< 65 or > 85	55(50)	28(88)	83(58)	
65–85	56(50)	4(13)	60(42)	
Albumin (g/L)				< 0.001
< 40 or > 55	61(55)	29(91)	90(63)	
40–55	50(45)	3(9)	53(37)	
AST (U/L)				< 0.001
< 15 or > 45	50(45)	28(88)	78(55)	
15–45	61(55)	4(13)	65(45)	
ALT (U/L)				< 0.001
< 9 or > 60	28(25)	19(59)	47(33)	
9–60	83(75)	13(41)	96(67)	
PA (mg/L)				< 0.001
< 250 or > 400	79(71)	29(91)	108(76)	
250–400	32(29)	3(9)	35(24)	
Urea (mmol/L)				0.652
< 2.9 or > 8.2	22(20)	10(31)	32(22)	
2.9–8.2	89(80)	22(69)	111(78)	
Uric acid (umol/L)				< 0.001
< 208 or > 428	36(32)	19(59)	55(38)	
208–428	75(68)	13(41)	88(62)	
Potassium (mmol/L)				0.318
< 3.5 or > 5.3	11(10)	6(19)	17(12)	
3.5–5.3	100(90)	26(81)	126(88)	
Sodium (mmol/L)				0.319
< 137 or > 147	36(32)	16(50)	52(36)	
137–147	75(68)	16(50)	91(64)	
Chlorine (mmol/L)				0.831
< 99 or > 110	24(22)	10(31)	34(24)	
99–110	87(78)	22(69)	109(76)	
PT (S)				0.052
< 9 or > 15	11(10)	2(6)	13(9)	
9–15	100(90)	30(94)	130(91)	
FIB (g/L)				< 0.001
< 2 or > 5	33(30)	24(75)	57(40)	
2.00–5.00	78(70)	8(25)	86(60)	

Abbreviations: Pre-blood transfusion means previous history of blood transfusion, Transfer means from a primary care facility to a hospital or from one hospital to another for a specific procedure or specialized treatment, T temperature, HR heart rate, MODS multiple organ dysfunction syndromes, WBC white blood cell, HGB hemoglobin, PLT platelets, NEU neutrophil count, AST aspartate aminotransferase, ALT alanine aminotransferase, PA prealbumin, PT prothrombin time, FIB fibrinogen.

### LASSO and multifactorial analysis

**Fig. 2:**
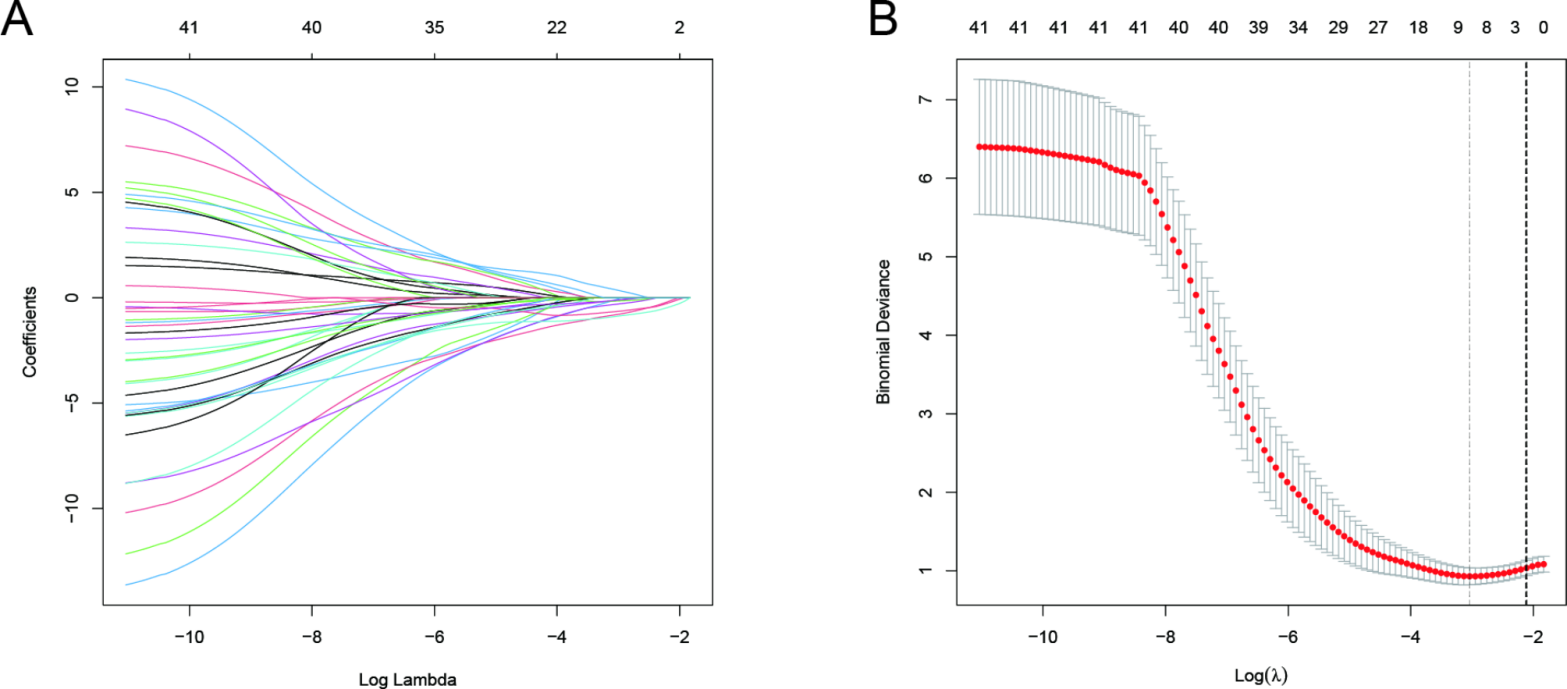
Demographic and clinical features of ACS selection using the LASSO binary logistic regression model. (**A**) Profiles of the LASSO coefficients for the 41 features. (**B**) Lambda selection utilized fivefold cross-validation with minimal criteria. Log versus partial likelihood deviance (binomial deviance) curve (lambda) Abbreviations: LASSO, least absolute contraction and selection operator; ACS, acute compartment syndrome

The LASSO regression model was employed to examine the data from both groups. The analysis revealed nine variables with significant differences, namely cause of injury, temperature, vascular damage, shock, total protein, AST, ALT, uric acid, and FIB. The distribution of variable coefficients and binomial deviation was plotted using LASSO binary logistic regression analysis (Fig. 2A and B). Furthermore, multivariate logistic analysis demonstrated that cause of injury, vascular damage, shock, and fibrinogen were independent risk factors for ACS amputation (p < 0.05, Table 2).

**Table 2:** Multivariate logistic analysis of risk factors for amputation in ACS.

Intercept and variable	Prediction model		
	*β*	Odds ratio (95% CI)	*P*-value
Intercept	3.358	28.74 (4.45-265.72)	0.001**
Cause injury	-2.315	0.10 (0.01–0.52)	0.013*
T	1.138	3.12 (0.63–16.42)	0.164
Vascular damage	-1.780	0.17 (0.05–0.52)	0.003**
Shock	-1.553	0.21 (0.04–0.90)	0.040*
Total protein	-0.842	0.43 (0.09–1.74)	0.251
AST	-1.097	0.33 (0.07–1.37)	0.143
ALT	-0.391	0.68 (0.19–2.36)	0.536
Uric acid	-0.742	0.48 (0.15–1.46)	0.193
FIB	-1.390	0.25 (0.07–0.77)	0.019*

**Note**: *β* is the regression coefficient. * p < 0.05, ** p < 0.01.

**Abbreviations**: ACS acute compartment syndrome, T temperature, AST aspartate aminotransferase, ALT alanine aminotransferase, FIB fibrinogen.

### Nomogram to predict the probability of amputation

**Fig. 3:**
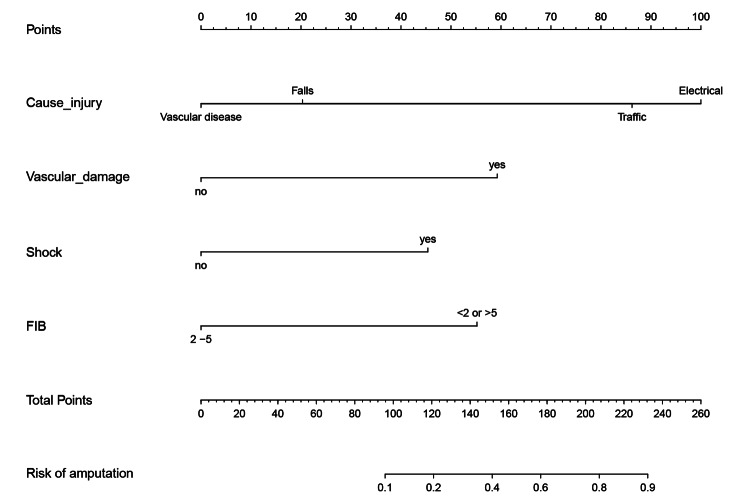
A nomogram based on independent risk factors for predicting amputation risk by calculating the total scores of the 4 parameters. FIB, fibrinogen

Based on the study results, several predictors were included in the model to estimate the probability of amputation in cases of injury. These predictors comprised the cause of injury (e.g., traffic injuries, falls, electric injuries, and vascular diseases), vascular damage to specific arteries (including the popliteal, anterior tibial, posterior tibial, brachial, radial, and ulnar arteries), hemorrhagic shock, and fibrinogen levels. Each predictor was assigned a corresponding score, and the total score was projected onto the bottom tier to create a nomogram (Fig. 3).

**Fig. 4:**
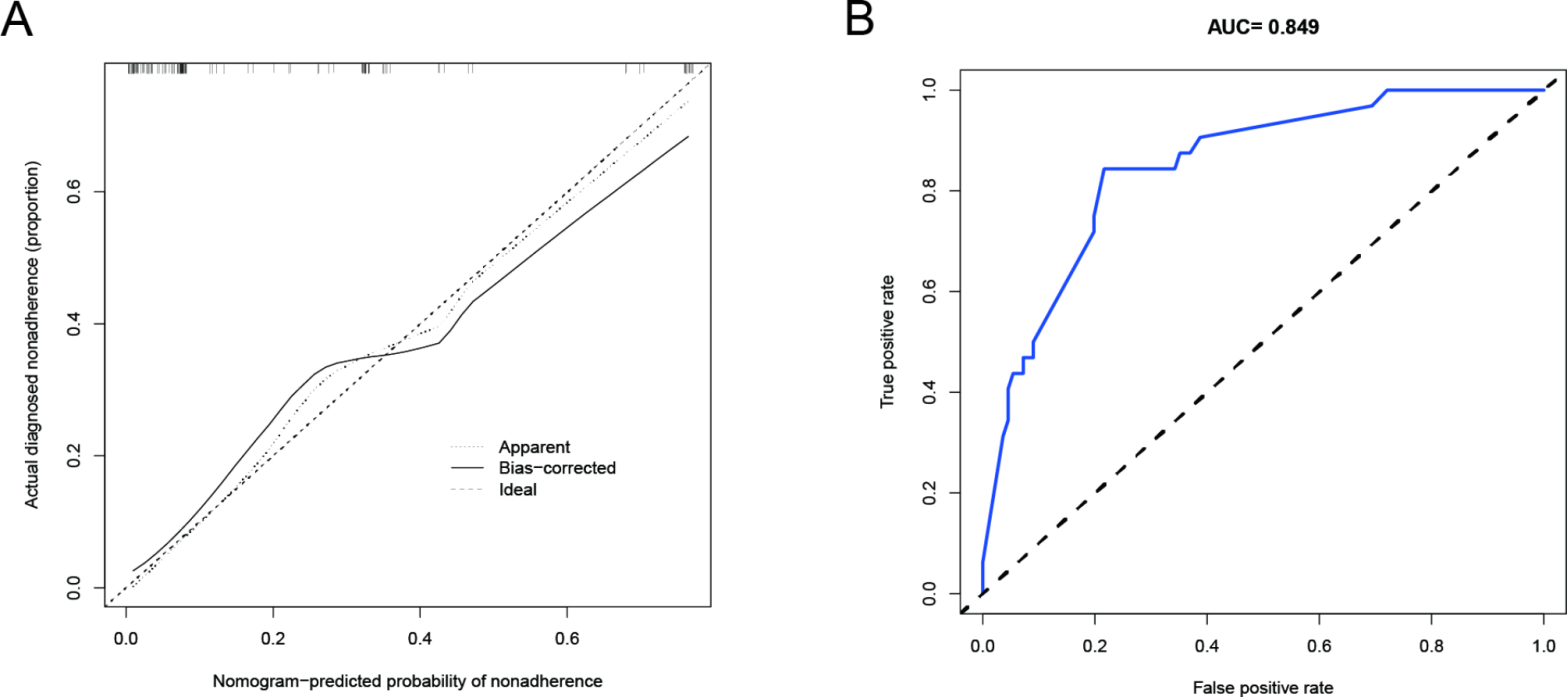
(**A**) Calibration curves of the nomogram in the cohort. (**B**) Comparison of the area under the receiver operating characteristic curve between nomogram-independent predictors

The predictive nomogram was assessed for calibration and discrimination using a calibration curve and an ROC curve. The calibration curve demonstrated that the nomogram was close to the ideal curve in the cohort, indicating a high degree of accuracy (Fig. 4A). The ROC curve was constructed with an AUC of 0.849, suggesting that the nomogram exhibited superior predictive power (Fig. 4B). The C-index of the predictive nomogram was 0.872 (95% CI: 0.854–0.962) in the cohort, which was determined by bootstrap validation to be 0.838, indicating good discriminatory power.

### Clinical application

**Fig. 5:**
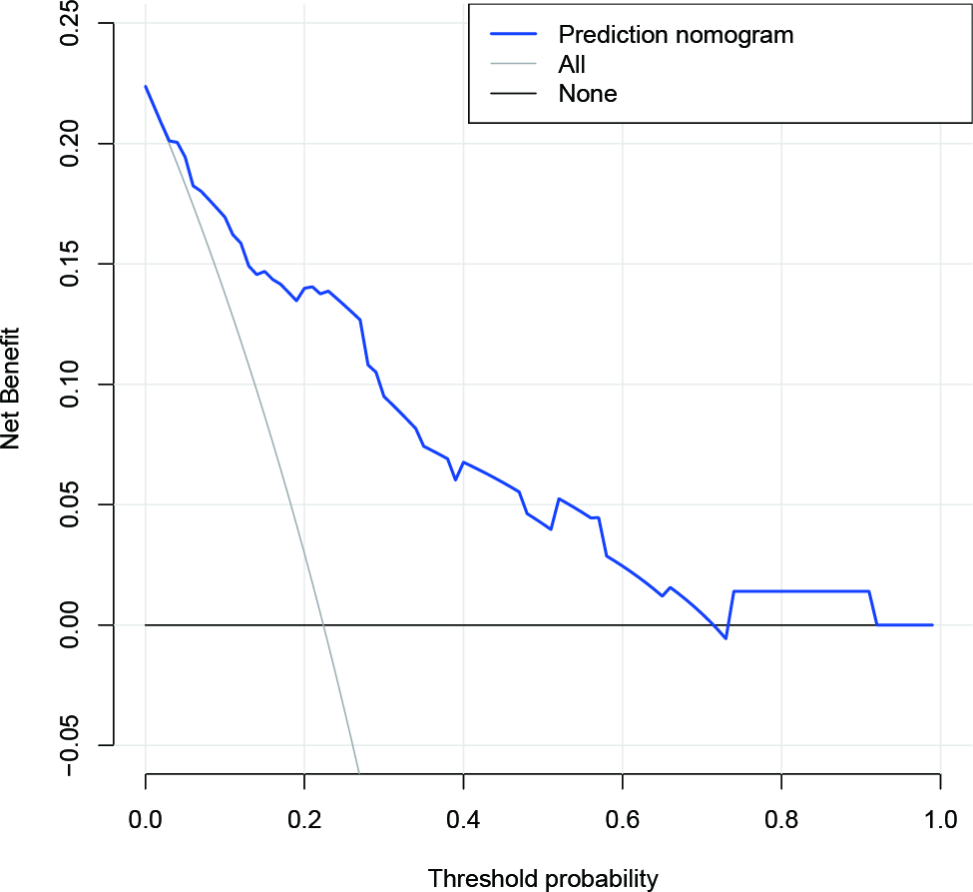
Decision curve analysis of the nomogram Notes: The net benefit is shown on the y-axis. The solid blue line shows the nomogram of amputation risk. The thin solid line shows that all patients are underwent amputation. The thick solid line shows that it is assumed that no patient will be underwent amputation. The decision curve showed that using this nomogram to predict amputation risk in the current study is better than the intervention-all-patients scheme or the intervention-none scheme when the threshold probability is 0.01 and 0.71, respectively

The decision curve analysis (DCA) of the amputation risk nomogram demonstrated its superior practicality and accuracy for risk thresholds ranging from 1 to 71% (Fig. 5). The DCA curve demonstrated that the amputation risk nomogram is a reliable tool for predicting the risk of amputation in patients with ACS.

## Discussion

Acute compartment syndrome (ACS) is a clinical condition that can cause limb-threatening and life-threatening consequences. Early diagnosis is challenging, and delayed fasciotomy decompression can lead to increased amputation rates and mortality [3, 17]. Additionally, the cost of care for ACS patients is high, with hospital costs three times higher and length of stay 2.3 times longer than patients without complications [18, 19]. Furthermore, delayed diagnosis and treatment can negatively impact surgeon and hospital reputations, with previous studies indicating that compensation is most commonly associated with these factors [20, 21]. As a result, identifying risk factors for amputation in ACS patients is crucial, as this can assist clinicians in identifying high-risk patients and managing them promptly to avoid adverse outcomes.

In this study, we developed a new predictive nomogram utilizing four easily available variables to determine the probability of amputation in patients with ACS. To analyze the risk factors associated with amputation, we retrospectively examined 143 patients with ACS. After adjusting for confounding factors, we included clinical characteristics and biochemical blood markers in the nomogram construction. The study identified independent risk factors associated with amputation, including cause of injury, vascular damage, shock, and fibrinogen, and developed a relatively accurate predictive nomogram for the risk of amputation. In internal sampling validation, the study showed high C-index and AUC values, demonstrating good discrimination and calibration of the nomogram.

Although causation has been recognized as a risk factor for acute compartment syndrome (ACS) in previous studies, it has not been identified as an independent risk factor for the development of amputation in ACS. Rhabdomyolysis occurs in about 10% of high-voltage electrical injury survivors due to massive skeletal muscle electrothermal conversion and electroporation [22]. Moreover, electrothermal injury can cause progressive muscle necrosis by inducing vascular thrombosis. Subsequently, the progressive tissue necrosis within the hard fascial sheath leads to progressive occlusion of the microcirculation, resulting in ACS [23]. Although muscle necrosis is not initially apparent, it can expand due to persistent ischemia, requiring staged debridement after initial decompression. Unfortunately, electrical burns often result in amputation [24–26], and our cohort’s findings confirm this. Therefore, ACS caused by electrical injury should receive greater attention in clinical practice and requires further investigation.

Previous studies have associated ACS incidence with massive blood loss, open fractures, and combined arterial injuries [27]. The rate of fasciotomy decompression in patients with peripheral vascular injuries is approximately 20.9–28%. Popliteal vascular injury patients have a particularly high treatment rate of 73.9% for fasciotomy decompression [28–30]. Among these patients, the amputation rate can be as high as 14% [27]. Patients with these injuries are often admitted in shock, which is an important prognostic factor for primary amputation on admission [31, 32]. Therefore, surgeons must remain highly vigilant to the risk of amputation due to ACS when these high-risk factors are present. Our study’s results support this caution and suggest that continuous physical examination, active pressure monitoring, and timely fasciotomy compression, along with vascular damage and antishock management, can help reduce the incidence of amputation in patients with vascular damage. In addition, previous studies have found that low albumin levels are an independent risk factor for amputation in patients with peripheral arterial disease [33]. This is consistent with our findings. Low albumin levels and malnutrition can lead to increased systemic inflammation and vascular sclerosis, and proper dietary supplementation can effectively prevent disease progression and worse outcomes [33–35]. Additionally, one study found that CPK levels were significantly higher in patients with confirmed compartment syndrome compared to those without the condition [36]. The authors concluded that measuring CPK levels could be a useful diagnostic tool in patients suspected of having compartment syndrome. However, it is important to note that elevated CPK levels can also occur in other conditions that cause muscle damage, such as trauma or exertional rhabdomyolysis [37]. Therefore, the specificity of CPK levels as a diagnostic tool for compartment syndrome is limited, and a high level of clinical suspicion and additional diagnostic tests (such as intracompartmental pressure measurements) are necessary for accurate diagnosis.

Fibrinogen plays a crucial role in the acute phase response following tissue injury, consisting of two phases: (1) thrombin cleavage products of fibrinogen with the inflammatory response to control tissue damage, stop blood loss, and prevent microbial infection, and (2) fibrinolytic products of fibrin and other matrix proteins with reparative inflammatory cells resulting in remodeling and repair of damaged tissue [38]. Fibrinogen levels have been shown to correlate with CRP levels, neutrophil and blood leukocyte counts, and independently predict the risk of amputation in diabetic foot ulcers, suggesting its potential utility in assessing disease severity and monitoring progression [39]. Additionally, fibrinogen may impact endothelial damage, fibrin clot formation, thrombosis, abnormal blood flow, and platelet hyperactivity. As a degradation product of fibrinogen, D-dimer reflects thrombus formation and breakdown and has been identified as an independent risk factor for radial artery occlusion [40, 41]. Our study demonstrated that fibrinogen is an independent risk factor for amputation in patients with ACS and deserves more attention.

Nevertheless, the present study has some limitations. Firstly, we included 143 ACS patients, of which 32 underwent amputation during hospitalization, resulting in a total amputation rate of 22.4%. It is important to note that our study may not have explored all possible reasons for amputation in trauma patients, and that other factors such as infection, ischemia, or severe soft tissue damage may also contribute to the decision to amputate. Secondly, while our nomogram’s accuracy was extensively verified through internal validation using bootstrap testing, external validation is lacking. The prevalence of ACS patients in other regions and countries remains unknown and warrants validation through large-scale, multicenter studies. Finally, our retrospective analysis covered an extended period, during which changes in surgical procedures and diagnostic techniques may have impacted ACS prognosis.

## Conclusions

Our study revealed that risk factors associated with amputation in ACS were highly correlated with injury etiology, vascular damage, shock, and fibrinogen levels. Additionally, we developed a new nomogram that shows excellent predictive ability to assist clinicians in making prompt and accurate predictions of amputation risk. Estimating the individual’s risk enables clinicians and patients to more comprehensively implement necessary measures to reduce the incidence of amputation by monitoring the condition and intervening medically.

## Data Availability

Due to institutional restrictions, the datasets collected and analyzed during the current investigation are not publically available but are available upon reasonable request from the corresponding author.

## Abbreviations

ACS: Acute compartment syndrome
ROC: Receiver operating characteristic
DCA: Decision curve analysis
AUC: Area under the curve
LASSO: Least absolute shrinkage and selection operator
HR: Heart rate
MODS: Multiple organs dysfunction syndromes
WBC: White blood cell
HGB: Hemoglobin
PLT: Platelets
NEU: Neutrophil count
AST: Aspartate aminotransferase
ALT: Alanine aminotransferase
PA: Prealbumin
PT: Prothrombin time
FIB: Fibrinogen
